# A Wearable Textile Thermograph

**DOI:** 10.3390/s18072369

**Published:** 2018-07-21

**Authors:** Pasindu Lugoda, Theodore Hughes-Riley, Rob Morris, Tilak Dias

**Affiliations:** 1 Advanced Textiles Research Group, School of Art & Design, Nottingham Trent University, Bonington Building, Dryden Street, Nottingham NG1 4GG, UK; tilak.dias@ntu.ac.uk; 2 School of Science and Technology, Nottingham Trent University, Clifton Lane, Nottingham NG11 8NS, UK; rob.morris@ntu.ac.uk

**Keywords:** electronic textiles, E-textiles, wearable electronics, smart textiles, temperature sensing, thermistor, wound management, sensor network, thermograph

## Abstract

In medicine, temperature changes can indicate important underlying pathologies such as wound infection. While thermographs for the detection of wound infection exist, a textile substrate offers a preferable solution to the designs that exist in the literature, as a textile is very comfortable to wear. This work presents a fully textile, wearable, thermograph created using temperature-sensing yarns. As described in earlier work, temperature-sensing yarns are constructed by encapsulating an off-the-shelf thermistor into a polymer resin micro-pod and then embedding this within the fibres of a yarn. This process creates a temperature-sensing yarn that is conformal, drapeable, mechanically resilient, and washable. This work first explored a refined yarn design and characterised its accuracy to take absolute temperature measurements. The influence of contact errors with the refined yarns was explored seeing a 0.24 ± 0.03 measurement error when the yarn was held just 0.5 mm away from the surface being measured. Subsequently, yarns were used to create a thermograph. This work characterises the operation of the thermograph under a variety of simulated conditions to better understand the functionality of this type of textile temperature sensor. Ambient temperature, insulating material, humidity, moisture, bending, compression and stretch were all explored. This work is an expansion of an article published in The 4th International Conference on Sensor and Applications.

## 1. Introduction

Treatment of chronic wounds in an aging population presents a growing concern in relation to health care budgets, accounting for £5.3 billion expenditure for the United Kingdom’s National Health service in 2015 alone [1]. The ability to continuously interrogate the wound during the healing process provides clinicians with enhanced treatment pathways with direct effects including prevention of amputations and reduction of hospitalisation time [2]. In recent years, the concept of wearable thermometry has gained significant interest for a number of potential medical applications such as detecting fever in patients [3], monitoring infants [4] and for health monitoring [5]. Some literature has proposed that localised (point measurements) temperature measurement could be used to detect diabetic foot ulcer formation [6,7,8]. Localised temperature measurement can also be used for wound care [9,10,11,12] as regional temperature changes at a wound site may indicate infection. Temperature is critical for normal cellular activity, and, therefore, the best condition for healing of wounds has been identified as 33 °C [12,13]. When the temperature of a wound falls below 33 °C, wound repair is hindered [13]. Quantitative measurements carried out by Fierheller et al. have found a relationship between the increased periwound temperature and wound infection [10]. As a result, a wearable thermograph capable of providing a remote temperature map of a wound would prove beneficial for clinicians when trying to manage wound care.

A wearable system for contact thermography has previously been developed by Giansanti et al. [14], and various other temperature sensors have been developed for wound temperature monitoring [15,16,17]. Most of these wearable temperature sensors are not manufactured on textile substrates, and, therefore, may prove to be uncomfortable to the end user. This is a particularly important consideration for a system that has to be in contact with delicate or damaged skin. One solution for creating a wearable temperature sensor is to create a temperature sensing electronic textile (E-textile). Textile fabrics are drapeable, comfortable and conformable. Previous work in the literature has developed textile-based temperature sensors by incorporating metal wires within the textile fabric [18,19,20,21]. A limitation with these sensors is that they are unable to provide localised temperature measurements, as a bulk measurement over the full area of the textile is taken. For a wearable thermograph the ability to provide spatially resolved temperature measurements is critical as wound infection would potentially occur over a small area only. This temperature change may not be correctly identified if a bulk temperature measurement is taken. Yarns embedded with micro-thermistors would provide an excellent base to create a textile base thermograph; a platform technology for embedding micro devices within the fibres of a yarn has been developed by Dias and co-workers [22,23]. This novel technology has been employed for a number of E-textile sensing applications [24,25] and temperature sensing yarn technology [23]; the temperature sensing yarns and their characterisation have been described in detail elsewhere [25].

An important aspect of this work is to fully understand the limitations of the textile thermograph. Most frequent errors in temperature measurement occur when trying to measure the surface temperature of a solid body in contact with a surrounding gas or liquid. The first partial error occurs as a result of the temperature sensor being in contact with the surface being measured. The sensor causes a more intense heat flow from the surface and this causes a drop in the surface temperature. The second partial error occurs due to the thermal contact resistance in-between the sensor and the surface; this is caused due to the non-ideal contact between the sensor and the surface. The third partial error occurs due to the sensor design resulting in an inaccurate reading [26]. This second partial error is due to the thermal contact resistance at the interface between the sensor and the surface being difficult to calculate, as the thermal contact resistance is influenced by the cleanliness and smoothness of the surface, the pressure the sensor is held on the surface, the elasticity of the surface, and the sensor [26].

Skin contact temperature measurement is also a complex process and is influenced by ambient temperature, sensor shape, contact of its sensing area to the surface (skin) and the thermal conductivity of the surface used to hold the sensor [27]. Installation will also have an effect on the temperature readings [28] and this will be particularly important for a textile-based sensor when the textile itself will have isolative properties. The effect of ambient temperature and installation type is often overlooked when measuring surface temperature, with only a few studies taking such effects into account [29,30,31]. Currently, there is no research on the effects of ambient temperature, fabric construction and other external conditions on temperature measurements by thermistors positioned within fabrics. It was also envisaged that a wound dressing made using temperature sensing yarn would be held in contact with the wound by placing a bandage on top of it, providing a layer of insulation. By covering the temperature sensing yarns with another fabric a microclimate around the skin can be created, potentially reducing the effects of ambient temperature on the temperature sensing yarn readings. An investigation by Buono and Ulrich [32] in various environmental conditions has shown that covered thermistors recorded higher skin temperatures when compared to when they were uncovered.

Sweat and other bodily fluids such as blood are also important factors that have to be taken into account when developing wearable sensors. Wounds are also generally moist and the moisture levels are known to be critical for wound healing [33]. Therefore, it is vital to understand the effects of moisture on the measurements of the temperature sensing yarn. It is also crucial to identify the effects of bending, compression and stretching on the temperature-sensing fabric. Cyclic tests conducted on the temperature-sensing yarn has shown that the temperature measurements of the yarn were not significantly affected by the cyclic tests and this has been presented in previous work [34].

The purpose of this work is to better understand how to take accurate skin temperature measurements using E-textiles produced with temperature-sensing yarns, and builds on the results of previous studies [25]. Initially, this work presents an optimised temperature-sensing yarn and identifies the effects on the absolute temperature measurements as a result of the braided sheath and packing fibres that comprise the yarn’s structure.

The work then looks at factors that would affect absolute temperature measurement when such a yarn is used for measuring skin contact temperature. A previous study done on the temperature-sensing yarn has demonstrated the importance of the temperature sensing yarn being in contact with the skin surface [35]. This study analyses the effects on the absolute temperature measured due to different heights of air gaps in-between the surface being measured and the yarn. Such a yarn would be incorporated within a textile fabric; therefore, the next set of experiments have been carried out to understand the effects of incorporating such a yarn in a knitted fabric. The effects of ambient temperature on the absolute temperature measurements are then investigated. The work also explores the effects of using different materials to cover the temperature-sensing fabric. Thereafter, it looks into the effects of humidity and moisture on the temperature sensor. Then, experiments have been conducted to identify the effects of bending, compression and stretching on the temperature-sensing fabric. This generates useful new knowledge for the community when investigating temperature sensing within textile structures.

This work finally presents a fully textile-based thermograph. The thermograph contains 16 temperature-sensing yarns of a type characterised in previous work [25,36], creating a fully conformable, drapeable, and washable temperature-sensing fabric.

This work is an expansion of an article published in The 4th International Conference on Sensor and Applications [37].

## 2. Materials and Methods

### 2.1. Yarn Fabrication

Murata 10 kΩ 100 mW 0402 SMD NTC thermistors (part number NCP15XH103F03RC, Murata, Kyoto, Japan) were used in this study to craft temperature-sensing yarns. In all cases, the thermistors were soldered onto copper interconnects (fine copper wires) as described in previous work [25].

**Figure 1:**
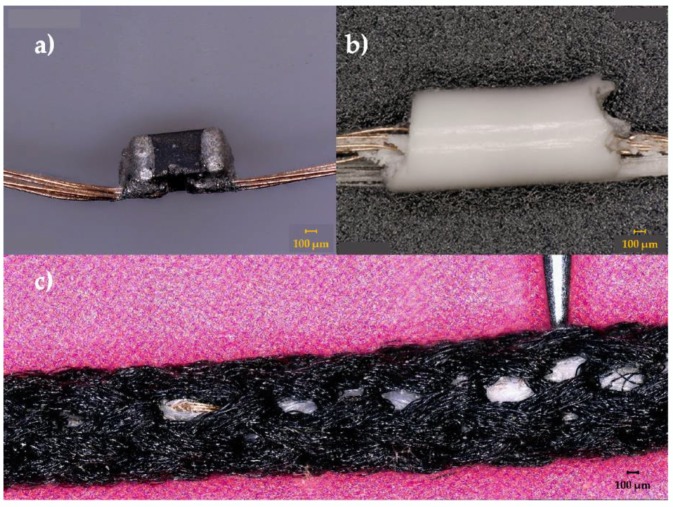
Photographs of prototype temperature-sensing yarn manufacturing process. (**a**) An eight-strand copper wire was soldered onto the thermistor; (**b**) the thermistor, copper wire and two polyester yarns were encapsulated with 9-20801 resin with 0.87 mm thickness; (**c**) the final yarn with the position of the thermistor indicated by a needle.

For the temperature-sensing yarns, two polyester yarns (167 dtex/48 filaments) were used as carrier fibres to provide mechanical strength to the copper wire interconnects and they were included within the encapsulation; the resin Multi-Cure^®^ 9-20801 (Dymax, Torrington, CT, USA) was used to form a cylindrical micro-pod with either a 0.87 mm or 1.53 mm diameter (a 0.87 mm diameter micro-pod was used unless otherwise stated). Thereafter, the carrier fibres, interconnects and the micro-pod were surrounded with six (167 dtex/48 filaments) polyester yarns (packing fibres in Figure 1) and a circular warp knitted structure (knit braid), which was produced with six additional polyester yarns (167 dtex/48 filament) to craft the temperature-sensing yarn. The various stages of a temperature-sensing yarn construction are shown in Figure 1 below.

Previous work has demonstrated that the accuracy of the temperature-sensing yarns was ±0.5 °C 63% of the time, or ±1 °C 89% of the time [25]. This was regarded as acceptable since the accuracy of the thermistor specified by the manufacturer for the inspected range (22.25–62.15 °C) was ±1.37 °C.

### 2.2. Experimental Setup

**Figure 2:**
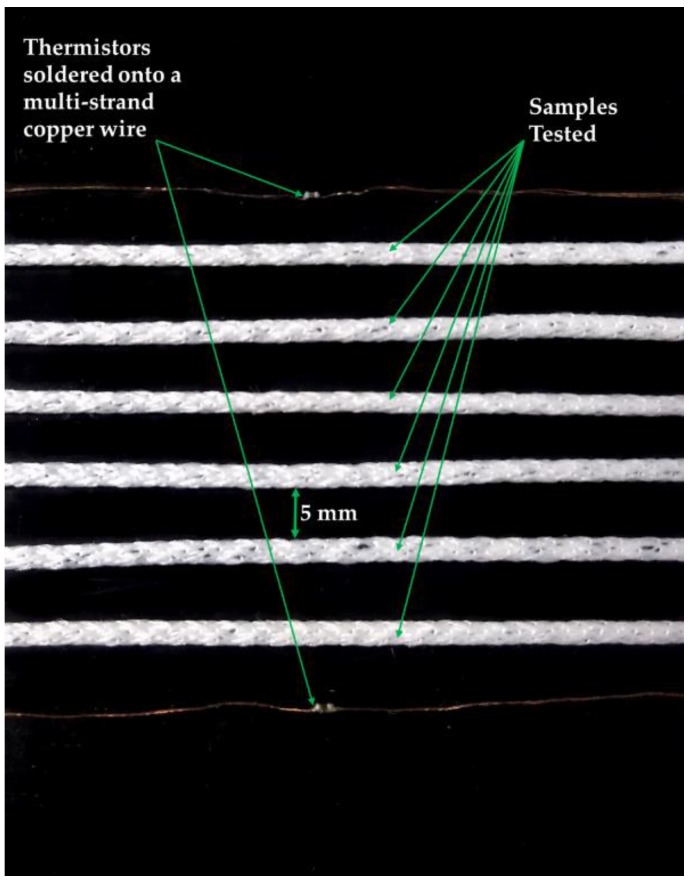
Photographs of the plate of the dry bath showing the positioning of the samples and the thermistors.

All experiments were conducted using an EchoTherm™ IC50 digital Chilling/Heating Dry Bath (Torrey Pines Scientific Inc., Carlsbad, CA, USA), henceforth known as dry bath. The samples being tested and two NTC thermistors (part number NCP15XH103F03RC, Murata, Kyoto, Japan) were secured onto the surface of the dry bath using insulating tape (RS Stock No.134-7319, RS, Corby, UK). The two thermistors were placed on either side of the samples on the dry bath in order to obtain the temperature of the plate of the dry bath. The samples and the two thermistors were positioned 5 mm from each other on the dry bath. The positioning of the samples and the thermistors are shown in Figure 2. A climate-controlled room was not used for most of the experiments, so room temperature was recorded using a k-type thermocouple (SE001, Pico Technology, St Neots, UK). The k-type thermocouple was positioned 40.0 cm to the right of the dry bath.

The samples and the thermistors were connected to potential divider circuits which contained a 10 kΩ resistor as the second resistor. The exact value of the second resistor was determined with an Agilent 34410A 6 ½ digital multi-meter (Agilent Technologies, Santa Clara, CA, USA) to a precision of 0.01%. The potential divider circuit was then connected to a data acquisition unit (NI DAQ USB 6008, National Instruments, Newbury, UK) and the thermocouple was connected to a PICO-TC08 unit (Pico Technology, St Neots, UK). Data was collected from both the NI-DAQ and the PICO-TC08 unit, and interpreted using a bespoke LabVIEW script (LabVIEW 2014 SP1, National Instruments™, Austin, TX, USA). Forty-two temperature readings were recorded every minute from each of the sensors (the samples tested, the two thermistors, and the two k-type thermocouples).

The temperature of the dry bath was increased in steps of 2 °C every 5 minutes. Previous work has shown that the temperature-sensing yarn has a step-response time of 0.17 ± 0.07 s for heating [25]. In order to ensure that a steady state was reached before capturing the temperature from each of the sensors, the average temperature for each of the temperature sensors (the thermistors and the samples) was calculated from the 84 readings recorded in between 3–5 min after changing the temperature of the dry bath. Thereafter, the average measurements from all the samples, the thermistors and the thermocouple have been plotted along with its 95% confidence intervals, in the results section.

### 2.3. Method to Analyse the Temperature Measurements

A way to analyse the data given in the paper is by identifying the measurement error. The significant difference between the true surface temperature and the temperature indicated by the sensor can be regarded as the measurement error. For these experiments, the true surface temperature was assumed to be the temperature captured by the thermistors positioned on the plate of the dry bath. The relationship between the true surface temperature and the indicated temperature can be defined by the equation given below [38].

(1) $$Z=\frac{T_{s}-T_{i}}{T_{s}-T_{a}}$$

where *Z* is the measurement error and *T_s_* is the true surface temperature, *T_i_* is the indicated temperature and *T_a_* is the temperature of the surrounding. This equation has been used to analyses results later in this work.

### 2.4. Understanding the Effects of Poor Contact between the Yarn and the Surface Being Measured

**Figure 3:**
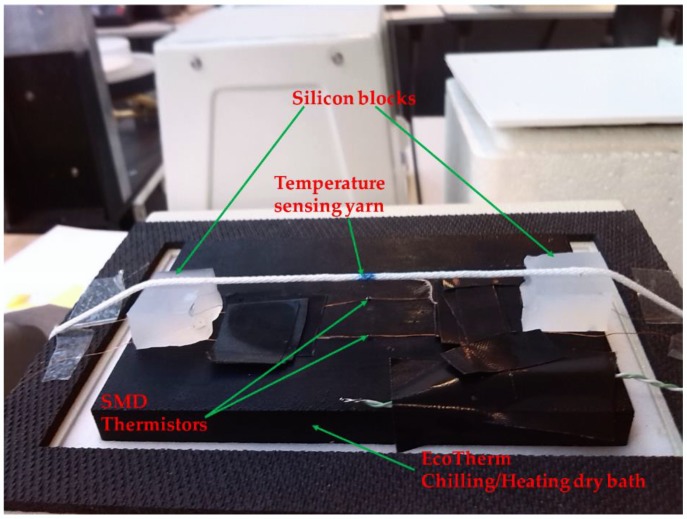
Photograph of the experimental setup used to investigate the effects of holding the temperature sensing yarn at different heights above the dry bath surface.

Poor contact between the wearer’s skin and a textile fabric leads to the formation of air gaps. The presence of an air gap between the wearer’s body and the textile could be mimicked by holding the temperature-sensing yarn at different heights above the surface of the dry bath. For this set of experiments the temperature sensing yarn was positioned 0.0 mm (directly on the plate), 0.5 mm, 1.0 mm, 2.0 mm, 5.0 mm and 10.0 mm above the plate of the dry bath. In order to achieve this, small blocks of silicon 10 mm by 10 mm with varying heights (0.5 mm, 1.0 mm, 2.0 mm, 5.0 mm and 10.0 mm) were positioned on the two edges of the dry bath. The temperature-sensing yarn was positioned on top of the silicon blocks and the sensing element (thermistor) of the temperature-sensing yarn was positioned in the middle of the dry bath (50 mm away from both of the silicon blocks). Figure 3 shows the experimental setup that was used for these experiments.

### 2.5. Temperature Sensing Yarn Positioned in a Textile Fabric

The temperature sensing yarn has to be knitted or woven into a fabric so that it can be applied as a wound dressing, or worn as a garment. Since the temperature-sensing yarn is currently produced in relatively modest quantities it was difficult to knit using the yarns, as large quantities are required for the knitting process. As a result, the temperature-sensing yarns were inserted into the textile structure post knitting, where 2.0 mm diameter channels were formed in fabrics during the knitting process, in order to insert the temperature-sensing yarn (as previously described [25]). This enabled positioning of the thermistors accurately to predetermined locations of the fabric. Using this technology, a fabric was knitted with eight channels positioned next to each other using a 3/357 d 100% white cotton yarn (Yeoman yarns, Liecester, UK) on a Stoll ADF332W E14 computerised flat-bed knitting machine (Stoll, Reutlingen, Germany), henceforth this fabric will be referred to as the temperature-sensing fabric. For these experiments, eight temperature-sensing yarns were positioned within the temperature-sensing fabric. The temperature-sensing fabric was secured onto the dry bath surface using an adhesive tape.

### 2.6. Understanding the Effects of Ambient Temperature

The temperature-sensing fabric when worn would be exposed to different ambient temperatures. Subsequently, it was important to investigate the effects of ambient temperature on the steady state error. Hence, the dry bath was set to 37 °C and placed inside of a S/SM-3200 benchtop environmental test chamber from Thermotron (Thermotron^®^, Holland, MI, USA). The environmental chamber temperatures was set to 7.01 ± 0.08 °C, 10.87 ± 0.07 °C, 20.60 ± 0.07 °C, 25.57 ± 0.05 °C, 30.23 ± 0.01 °C, 39.88 ± 0.10 °C, 49.97 ± 0.09 °C and held for 10 min to ensure that a steady state temperature was reached. The relative humidity was set to 60% (which is the humidity recorded in London during the summer). It should be noted that increasing the humidity beyond 60% resulted in water droplets forming on the plate surface for the higher temperatures tested. Two bare thermistors and the temperature-sensing fabric containing eight temperature sensing yarns were examined.

### 2.7. Reducing Ambient Temperature Effects by Covering the Fabric with Different Materials

**Figure 4:**
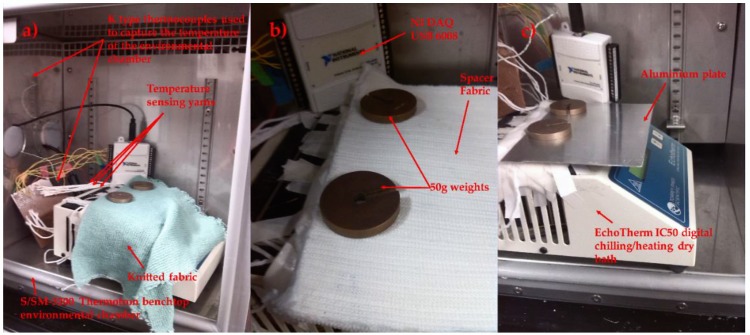
The eight temperature sensing positioned within the temperature sensing fabric covered various materials. (**a**) A knitted fabric; (**b**) a knitted spacer fabric; (**c**) an aluminum plate.

It was envisaged that the temperature-sensing fabric when used as a wound dressing would be covered by a bandage. The bandage would impede the heat flow between the thermograph and external environment, since it is known that insulating a temperature sensor impacts the temperature captured by the sensor [32]. Therefore, three different materials with varying thermal conductivities were chosen to cover the temperature-sensing fabric containing the eight temperature-sensing yarns; a knitted fabric, a knitted spacer fabric and an aluminium plate. The knitted fabric was a 1 × 1 rib structure knitted using an acrylic 2/28 nm yarn (1-ply) (Uppingham Yarns, Uppingham, UK) using the Stoll ADF 3 32W E14 knitting machine (STOLL, Reutlingen, Germany), henceforth it will be referred to as the knitted fabric. The spacer fabric was knitted using 167 dtex/48 filament polyester yarn (J H Ashworth & Son Ltd., Manchester, UK) using a STOLL CMS822HP E16 computerised flat-bed knitting machine (henceforth referred to as the spacer fabric). The thickness of the knitted fabric and the spacer structure was measured at 2.2 ± 0.1 mm and 5 ± 0.1 mm respectively. The thickness of the aluminium plate used to cover the temperature-sensing fabric was measured to be 0.99 ± 0.02 mm. The covering materials were held in place using two 50 g weights at the edges of the material. The experimental set up for the three covering materials is displayed in Figure 4. As previously described, the dry bath was placed inside of the environmental chamber with a fixed environmental temperature of 25.51 ± 0.20 °C. A range of dry bath temperatures between 20–40 °C were investigated at 2 °C temperature increments.

### 2.8. Effects of Humidity on the Temperature Sensing Fabric

The temperature-sensing fabric was tested at different levels of ambient humidity to validate its operation in different climates. In order to do this, the environmental chamber was used, the relative humidity of the environmental chamber was changed from 30–80% in steps of 10%. The dry bath temperature was kept constant at 34.38 ± 0.35 °C and the environmental chamber temperature was held constant at 25.60 ± 0.43 °C.

### 2.9. Identifying the Effects of Moisture on the Temperature Sensing Fabric

**Figure 5:**
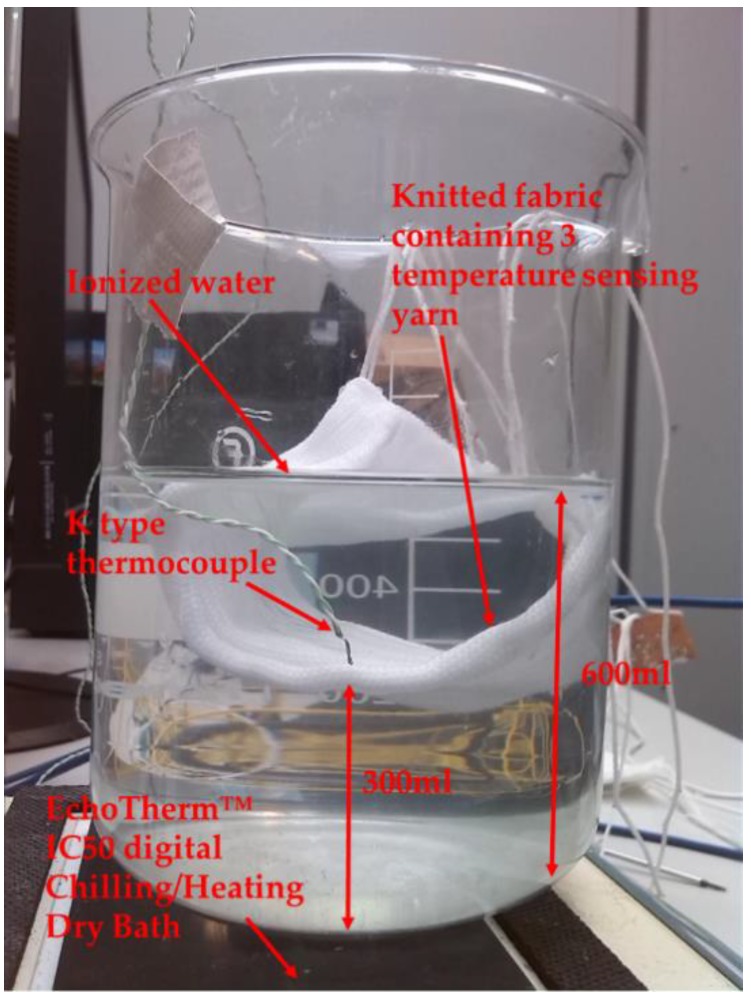
Experimental setup to measure temperature from the Beaker containing Ionised water.

The moisture experiments were conducted using ionised water since using bodily fluids is beyond the scope of this work; however, in the future these temperature sensors will be tested using bodily fluids. Initially, a preliminary experiment was conducted by positioning a temperature-sensing fabric containing three temperature-sensing yarns in a beaker of ionised water. Even though submerging the fabric within a beaker of water can be considered an extreme example, it provides a clear overview as to the performance of the temperature-sensing yarns with regards to moisture. For this experiment, the beaker was filled with 600 mL of ionised water and the temperature-sensing fabric containing the temperature-sensing yarns and a k-type thermocouple (SE001) were positioned 300 mL below the surface of the water as shown in the Figure 5.

For this experiment, the beaker containing ionised water was heated using the EchoTherm™ IC50 digital Chilling/Heating Dry Bath and the temperature captured by the k type thermocouple and the temperature-sensing yarns were recorded. The dry bath temperature was set to 100 °C in order to heat the water in the beaker. The beaker was left on the dry bath for 10 min in order to ensure the temperature distribution throughout the water was uniform. Then the measurements from the temperature-sensing yarns and the thermocouples were captured using the experimental setup discussed in Section 2.2.

#### Experiment to Understand the Effects of Absorption and Evaporation

A more practical experiment was conducted to identify the effects of moisture on the temperature-sensing yarns, using a Gravimetric Absorbing System from MK Systems (Model MK 251, M/K GATS, Peabody, MA, USA). The M/K GATS system accurately measures liquid absorption rates and total capacity. The M/K GATS system has been designed to comply with ISO 9073-12:2002 [39]. The temperature-sensing fabric containing the three temperature-sensing yarns, which was used in the preliminary experiment, was also used for this experiment. Two k type thermocouples (SE001) were inserted within two channels of the temperature-sensing fabric on either side of the three temperature-sensing yarns. The temperature-sensing fabric was positioned on top of the porous plate of the M/K GATS system and the temperature measurements were recorded when the water was being absorbed by the temperature-sensing fabric. To make the experiments more realistic, the temperature of the ionised water used in the M/K GATS system was heated to 36.2 °C, this was measured using a k-type thermocouple (SE001) positioned in the lower reservoir of the M/K GATS system. The room temperature during the experiment was measured at 22.7 ± 0.3 °C using another k type thermocouple (SE001).

**Figure 6:**
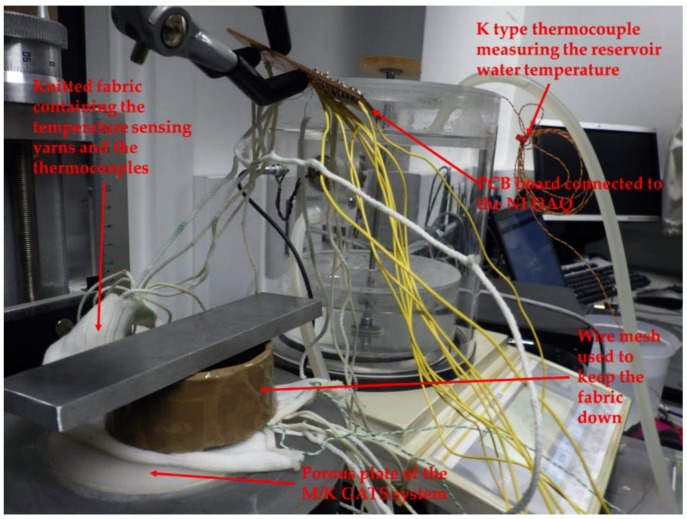
The experimental setup used for the moisture absorption experiment using the M/K GATS system.

Since the temperature-sensing yarns had to be connected to the NI DAQ, special care had to be taken to prevent the water from flowing into the interface circuit; therefore, a stand was used to position the interface circuit above the porous plate. This in turn effected the contact between the porous plate and the temperature-sensing fabric. One solution to enhance the contact in-between the porous plate and the fabric was to use a wire mesh on top of the fabric [40]; however, the weight of the wire mesh was insufficient to hold the temperature-sensing fabric in place. Therefore, a weight of 119.17 g measured using an Adam balance (PW 214 Adam equipment PW analytical balance, Adam Equipment Ltd., Milton Keynes, UK) was placed on top of the wire mesh as shown in Figure 6.

The amount of water absorbed was measured using the software MK systems GATS 3.4.0 provided by M/K systems. Once the temperature-sensing fabric reached saturation, the temperature-sensing fabric was taken away from the porous plate and left to dry. The temperature measurements were recorded from the temperature-sensing yarns and the thermocouples when the water was being absorbed by the temperature-sensing fabric and when the temperature-sensing fabric was left to dry.

### 2.10. Identifying the Effects of Bending, Compression, and Stretching on the Temperature Sensing Fabric

**Figure 7:**
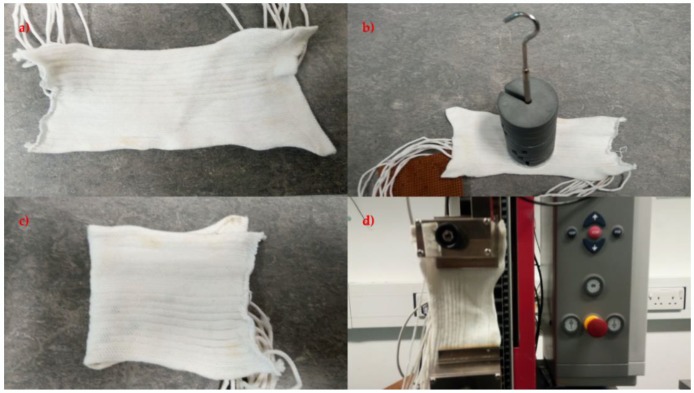
Displays the four preliminary experiments conducted on the temperature-sensing fabric where (**a**) the fabric was left to rest, (**b**) a weight of 1 kg was used to compress the fabric, (**c**) the fabric was bent and (**d**) the fabric was stretched using the Z 2.5 Zwick/Roell tensile testing machine.

The temperature-sensing fabric containing the eight temperature-sensing yarns (presented in Section 2.5) was also used for these experiments. Four preliminary experiments were conducted to identify the effects of bending compression and stretch on the temperature-sensing fabric. The first experiment was done on the temperature-sensing fabric where the temperature measurements from the temperature sensing yarns were obtained when the fabric was left to rest on a table. The next experiment was carried out to identify the effects of compression, to achieve this a weight of 1 kg was placed on top of the temperature sensors in the temperature-sensing fabric. Then an experiment was conducted to understand the effects of bending where the fabric was bent at the location of the temperature sensors in the fabric. Finally, the fabric was stretched by 5% using a tensile testing machine (Z2.5, Zwick/Roell, Ulm, Germany). All these experiments were conducted at room temperature which was recorded using a k type thermocouple (SE001) at 26.9 ± 0.3 °C. The Figure 7 presents the temperature-sensing fabric during the four different experiments.

### 2.11. Prototype Textile Thermograph

A prototype thermograph was created using 16 temperature-sensing yarns. The thermograph was constructed as a spacer fabric with 16 channels for positioning the temperature-sensing yarns. A spacer fabric was chosen since most of the commercially available wound dressings have a similar construction. The spacer fabric was also knitted on the Stoll ADF 3 32W E14 machine (Stoll, Reutlingen, Germany) using three polyester 167/48 dtex yarns (J H Ashworth & Son Ltd., Manchester, UK) twisted together (henceforth this will be referred to as the textile thermograph). This textile thermograph was made using polyester for demonstration purposes; however, these temperature-sensing yarns and the thermograph could be made using cotton/polyamide fibres, which are generally used to create wound care products (such as bandages, wadding) [41]. The circular warp knitted structure (knit braid) that covers the cylindrical micro-pod and the copper filaments in the temperature-sensing yarn, ensures that only the textile fibres are in contact with the surface of the skin/wound.

**Figure 8:**
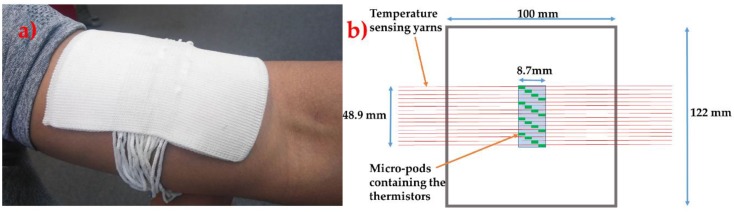
(**a**) A photograph of the textile thermograph. (**b**) A schematic of the textile thermograph.

The wearable textile thermograph had dimensions of 122 mm by 100 mm and thickness of 3.36 ± 0.02 mm. The temperature-sensing yarns were positioned 1.157 ± 0.023 mm apart from one another (measured using VHX Digital Microscope, Keyence, Milton Keynes, UK). The micro-pods thermistors in the temperature-sensing yarn were positioned in the middle of the textile thermograph one after the other diagonally as shown in Figure 8. This textile thermograph provides a 2-dimensional map of the surface being measured; however, positioning the temperature-sensing yarns one after the other causes an offset of 10.5 ± 0.023 mm in the y direction. The offset can be eliminated by positioning several thermistors within a single temperature-sensing yarn. This can be achieved by soldering a SMD thermistor (part number NCP15XH103F03RC) onto enamelled (insulated) copper wire and positioning several of them within a single temperature-sensing yarn. Future work will look into this and identify the effects of using enamelled copper wires instead fine copper wires.

The interface hardware comprised of a 16 channel multiplexer (Analog/Digital MUX Breakout-CD74HC4067, SparkFun Electronics, Niwot, CO, USA), an Arduino Pro Mini (Arduino, Turin, Italy) and a SparkFun Bluetooth Mate Gold (SparkFun Electronics, Niwot, CO, USA). Initially the temperature-sensing yarns were soldered onto male header connectors and the solder joints were protected using heat shrinkable sleeves. The male headers were then connected onto the 16-channel Analog Multiplexer. The four digital input channels of the multiplexer were connected to the digital output of the Arduino Pro Mini. The signal output from the multiplexer was connected to the analogue input of the Arduino Pro Mini. A 10 kΩ resistor was used to ground the analogue input of the Arduino Pro Mini (the 10 kΩ resistor was used as the load resistor to complete the potential divider circuit). The Arduino Pro Mini was also connected to a SparkFun Bluetooth Mate Gold which provided the Bluetooth connectivity to the wearable textile thermograph. LabVIEW (version 14.0.1, Austin, TX, USA) was used to program and create a graphical user interface. A photograph of the textile based wearable textile thermograph, alongside a schematic, is shown in Figure 8.

## 3. Results

### 3.1. Identifying the Effects of the Fabrication of the Yarn on the Temperature Measurements of the Thermistor

#### 3.1.1. Effect of Using Thermally Conductive Resin to Form Micro-Pod

**Figure 9:**
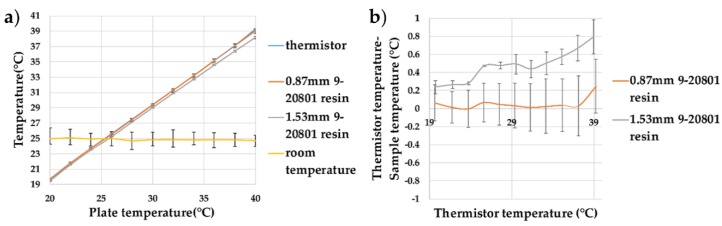
Thermistor samples encapsulated within resin micro-pods created with thermally conductive (9-20801) resin. (**a**) The effect of increasing the plate temperature on the temperature recorded by the thermistor and the samples (0.87 mm 9-20801 resin and 1.53 mm 9-20801 resin); (**b**) the difference between the temperatures recorded by the samples compared to the thermistor temperature, showing the deviations from the expected values. The lines shown in the graphs are not a data fitting but intended as a guide for the eye. The error bars shown in the figure represent 95% confidence intervals.

Initially, experiments were conducted to refine the temperature-sensing yarn design presented in previous work. Unlike in earlier work, here it was important to obtain an absolute temperature measurement. Here, encapsulated thermistors using thermally conductive resin micro-pods were investigated (in previous work [25] a non-thermally conductive resin was used). Two sizes of resin micro-pod were studied with the temperatures varied from 20 to 40 °C. This range covered the variation in skin temperature which was approximated to 25–38 °C in the literature [42]. The recorded temperature as a function of the dry bath temperature, and the difference in the recorded and actual temperatures, have been shown in Figure 9.

When using the thermally conductive (9-20801) polymer resin, the thermistor within 0.87 mm diameter cylindrical encapsulation showed very little deviation from the bare thermistor measurements (the trend for the thermistor temperature—sample temperature would give −0.09 °C deviation at 0 °C and a +0.39 °C at 100 °C). When a thermistor was encapsulated within a 1.53 mm diameter micro-pod, a measurement error of 0.05 ± 0.057 was recorded. A clear trend could be observed between the temperature recorded by the thermistors on the dry bath and the sample measurements; the trend for the (thermistor temperature—sample temperature) showed −0.27 °C deviation at 0 °C and a +2.27 °C at 100 °C. This was due to the increase in the thermal resistance of the resin at the greater volume used for the 1.53 mm diameter micro-pod. As the 0.87 mm 9-20801 resin micro-pod gave the least variation in absolute temperature, this micro-pod design was used to produce a set of temperature-sensing yarns.

#### 3.1.2. Identifying the Effect of the Packing Fibres and the Circular Warp Knitted Structure (Knit Braid) on the Temperature Measurements

**Figure 10:**
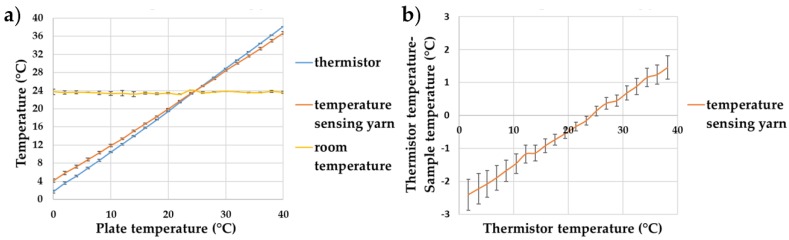
Temperature sensing yarns created using thermally conductive (9-20801) resin micro-pods. (**a**) The effects of increasing the plate temperature on the temperature recorded by the thermistor and the temperature-sensing yarns; (**b**) the difference between the temperatures recorded by the samples compared to the thermistor temperature. The lines shown in the graphs are not a data fitting but intended as a guide for the eye. The error bars shown in the figure represent 95% confidence intervals.

The final temperature-sensing yarn was made by covering the micro-pod with packing fibres and a circular warp knitted structure (knit braid), which was produced with six additional polyester yarns. Subsequently, the temperature-sensing yarns, created using 0.87 mm thermally conductive (9-20801) resin micro-pods, were tested over a temperature range of 0–40 °C to validate their operation. The results are shown in Figure 10.

Figure 10b shows that the packing fibres and the braided yarn layer impact the absolute temperature measurements of the temperature-sensing yarn, with a significant differences recorded in the absolute temperature compared to the temperature recorded for the bare thermistor; the trend for the (thermistor temperature—sample temperature) would give −2.60 °C deviation at 0 °C and a +8.09 °C at 100 °C. The difference between the temperature of the thermistor and the temperature-sensing yarn is almost zero at room temperature; it is interesting to note that the difference at physiologically relevant temperatures are still fairly minimal (~1 °C).

The measurement error caused due to the packing fibres and the braided yarn can be calculated as 0.12 ± 0.006 using Equation (1). Therefore, by using this value and Equation (1), the actual temperature of the surface can be obtained from the temperature captured by the temperature-sensing yarn.

### 3.2. Understanding the Effects of Positioning Temperature Sensing Yarn at Different Distances above the Surface Being Measured

**Figure 11:**
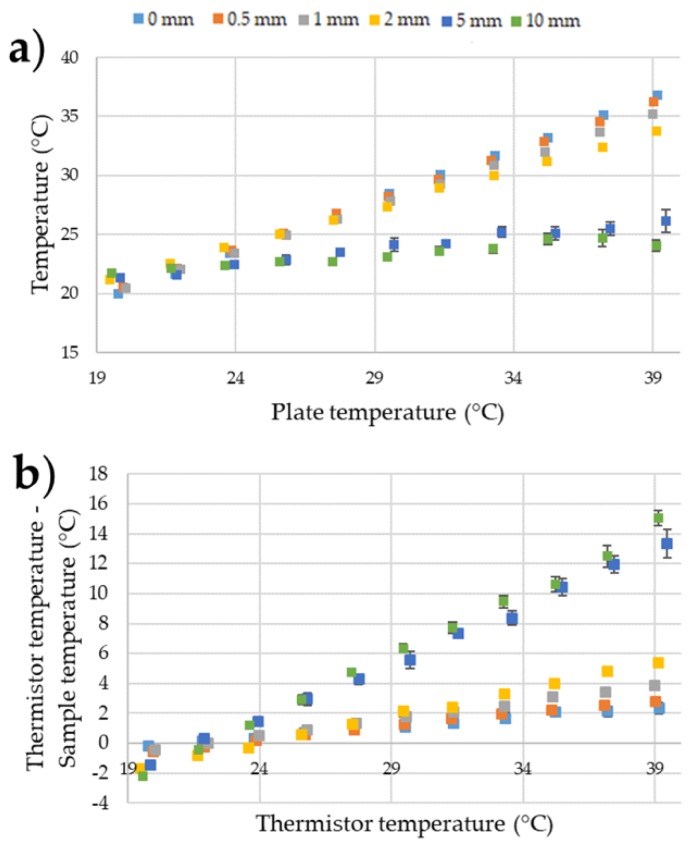
(**a**) Temperature measurements of the temperature-sensing yarn when it is held at different heights above the surface of the dry bath; (**b**) the difference between the temperatures recorded by the temperature-sensing yarns compared to the thermistor temperature. The error bars shown in the figure represent 95% confidence intervals.

It was important to identify the impact of poor contact (i.e., non-direct contact) between the temperature-sensing yarn and the surface being measured. The results given in Figure 11 illustrate the effects of holding the temperature sensing yarn at different heights above the surface of the dry bath.

**Figure 12:**
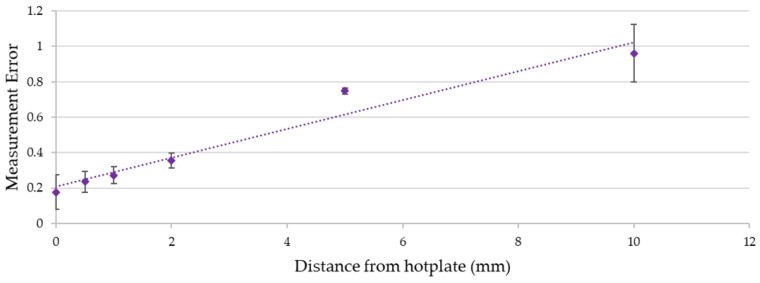
The relationship between the measurement error (calculated using Equation (1)) and the distance of the temperature-sensing yarn from the surface of the dry bath. The dotted line shows a linear data fitting. The error bars shown in the figure represent 95% confidence intervals.

It can be observed from the results in Figure 11 that keeping the temperature-sensing yarn at different heights above the surface of the dry bath increased the measurement error. Figure 12 below gives the relationship between the distance of the temperature sensing yarn from the surface of the dry bath and the measurement error.

It can be observed from Figure 12 that the measurement error increases almost linearly with the increase in the distance from the dry bath surface. This effect can be related to Fourier’s law of heat conduction given in Equation (2) where the difference in temperature (∆*T*) between the temperature-sensing yarn and the dry bath surface is directly proportional to the distance in-between them (∆*x*).

∆*T* = −(∆*x* × *Q_Cond_*)/(*k* × *A*) (2)

where *k* is the thermal conductivity of air, *A* is the area of which heat is being transferred and *Q_Cond_* is the rate of heat conductance [43]. Equation (2) neglects heat transfer through convection and radiation and assumes that the air in-between the dry bath surface and the temperature-sensing yarn is static with no significant convection occurring. A further assumption made was that the white fibres and the white micro-pod made the temperature sensing yarn reflective to the infra-red spectrum, prohibiting radiative heat transfer. White reflective surfaces are known to have a low absorption and emission of radiant heat [44,45].

Even when the temperature-sensing yarn was kept 0.5 mm away from the dry bath, the measurement error increased to 0.24 ± 0.03. Therefore, it was crucial to ensure that any garment knitted or woven using temperature-sensing yarn was held in contact with the skin surface when taking temperature measurements. A similar conclusion was drawn during a user trial conducted using temperature-sensing socks in earlier work [35]. It was envisaged that when the temperature-sensing yarn is used as a wound dressing that it will be held in contact with the skin or a wound by the bandage wrapped around the wound dressing. Bandaging would therefore have to be tight to reduce the distance between the thermograph and the wound to a minimum to ensure the most accurate results.

### 3.3. Temperature Sensing Yarn Positioned in a Knitted Fabric

**Figure 13:**
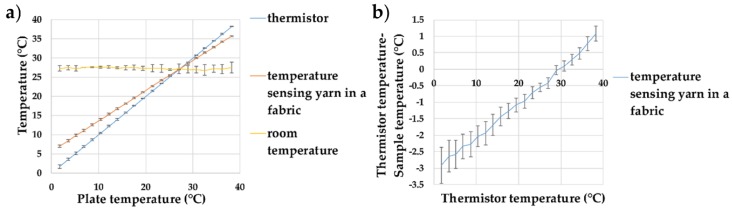
(**a**) Temperature measurements taken from the temperature-sensing yarns positioned within the temperature-sensing fabric; (**b**) the difference in-between the temperature recorded by the temperature-sensing yarn in the temperature-sensing fabric compared to the thermistor temperature. The lines shown in the graphs are not a data fitting but intended as a guide for the eye. The error bars shown in the figure represent 95% confidence intervals.

A temperature-sensing fabric containing the eight temperature-sensing yarns was fully characterised over a range of temperatures from 1.71–38.19 °C using the dry bath (here only a single, point measurement was taken). The temperature given by the temperature-sensing yarns in the temperature-sensing fabric was compared to the temperature of the dry bath captured using the thermistors and is shown in Figure 13.

Figure 13 demonstrates that when the temperature-sensing yarn was placed inside the knit fabric it increased the steady state error. The measurement error increased to 0.24 ± 0.06. This was the same error as holding the yarn 0.5 mm away from the dry bath (as observed above). Therefore, this effect was likely due to a contact error created by the temperature-sensing yarn not being in direct contact with the dry bath.

### 3.4. Understanding the Effects of Ambient Temperature

**Figure 14:**
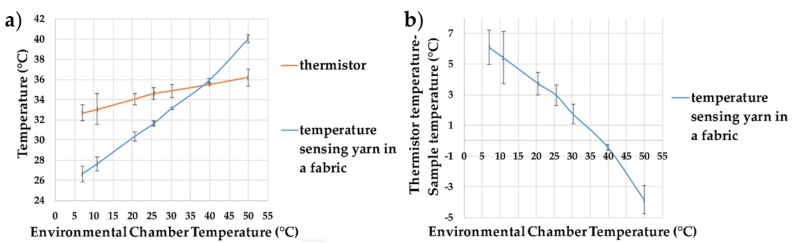
(**a**) Temperature measurements from the temperature sensing yarns positioned within the temperature sensing fabric at different ambient temperature; (**b**) the difference between the temperature recorded by the temperature sensing yarn in the temperature sensing fabric compared to the thermistor temperature. The lines shown in the graphs are not a data fitting but intended as a guide for the eye. The error bars shown in the figure represent 95% confidence intervals.

Experiments were conducted to identify the effect of ambient temperature on the temperature captured by the temperature-sensing yarns positioned within the temperature sensing fabric, with results presented in Figure 14. The experiments were carried out in the environmental chamber (previously described). A minimum temperature of 7.01 ± 0.08 °C was explored as the chamber was unable to reach lower temperatures under these experimental conditions. At 49.97 ± 0.09 °C, water droplets formed on the surface of the dry bath. This effected the readings of the two thermistors soldered onto copper wires. Therefore, the measurements at 49.97 ± 0.09 °C were taken after the first 5 min. Figure 14a gives the environmental chamber temperature against the temperature measured by the thermistors and the temperature-sensing yarns.

It can be observed from Figure 14a that the ambient temperature has an effect on the measurements of both the thermistor and the temperature-sensing yarn. The ambient temperature effects the temperature-sensing yarn more than the thermistor, which is likely due to the greater surface area of the temperature-sensing yarn (allowing for a greater heat transfer). By using Equation (1) the measurement error can be calculated as 0.26 ± 0.03, which is similar to the measurement error obtained (0.24 ± 0.06) when the temperature-sensing fabric was placed at room temperature and the dry bath surface temperature was changed (given in Figure 13). This proved the impact of ambient temperature on surface temperature measurement.

### 3.5. Reducing Ambient Temperature Effects by Covering the Fabric with Different Materials

**Figure 15:**
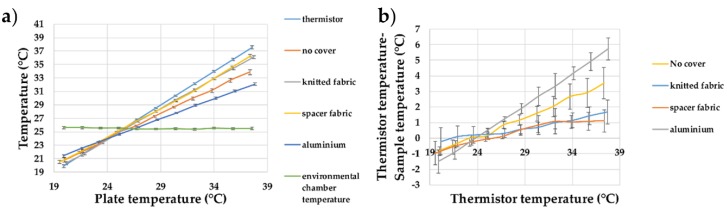
(**a**) Temperature measurements from the temperature-sensing yarns positioned within the temperature-sensing fabric when it is covered using different materials; (**b**) the difference between the temperature-sensing yarn measurements and the thermistor measurements. The lines shown in the graphs are not a data fitting but intended as a guide for the eye. The error bars shown in the figure represent 95% confidence intervals.

The effects of covering the temperature-sensing fabric containing the eight temperature-sensing yarns with different materials have been studied. The thermal conductance of the knitted fabric and the spacer fabric was determined using the experimentation demonstrated in the literature [46] with the knitted fabric = 0.074 Wm^−1^k^−1^; the knitted spacer fabric = 0.051 Wm^−1^k^−1^; and the aluminium plate = 205 Wm^−1^k^−1^ [47]. Figure 15 shows the recorded temperature from the temperature-sensing fabric compared to the dry bath surface temperature for the different materials which are used as covers.

It can be seen that covering the temperature sensing with an insulator (knitted fabric having a thermal conductivity of 0.074 Wm^−1^K^−1^ or a spacer structure having a thermal conductivity of 0.051 Wm^−1^K^−1^) reduced the measurement error, whereas covering it with a thermally conductive material (aluminium having a thermal conductivity of 205 Wm^−1^K^−1^) increased it, which would be expected from Fourier’s law of heat conduction (given in Equation (2)). The thermal conductivity of the material used to cover the temperature sensing fabric was proportional to the heat transfer from the textile temperature-sensing fabric containing the temperature-sensing yarns, to the surrounding. By increasing the thermal conductivity of the covering material, the heat flow from the temperature-sensing fabric to the surroundings would increase, assuming all the other variable remain constant; this increased the measurement error. When the textile thermograph would be used in wound dressings, the covering bandage wrapped around the dressing would provide insulation to the textile thermograph.

### 3.6. Effects of Humidity on the Temperature Sensing Yarns Positioned within the Temperature Sensing Fabric

**Figure 16:**
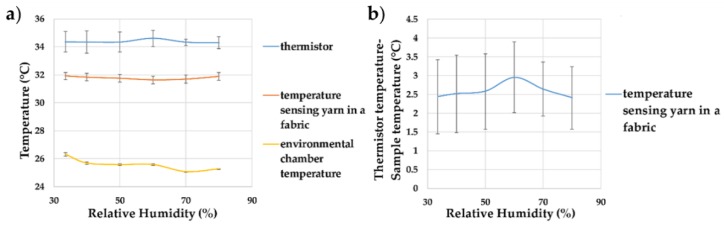
(**a**) Effects of relative humidity on the temperature measurements of the temperature-sensing yarn (the average temperature recorded by the eight temperature-sensing yarn has been plotted) in the temperature sensing fabric; (**b**) the difference between the temperature-sensing yarn measurements and the thermistor measurements at different humidities. The lines shown in the graphs are not a data fitting but intended as a guide for the eye. The error bars shown in the figure represent 95% confidence intervals.

For completeness, the next set of experiments were carried out to understand the impact of humidity on the temperature recorded by the temperature-sensing yarns positioned within the temperature-sensing fabric. Relative humidity levels between 30–80% were explored, as shown in Figure 16.

Figure 16 showed that humidity did not affect the recorded temperature significantly, with the recorded temperature holding at 31.80 ± 0.35 °C over all changes of the relative humidity in the environmental chamber. This would be expected given the waterproofing on the temperature-sensing elements and the small (relative) volume of fibres in which moisture could become trapped.

### 3.7. Effects of Moisture on the Temperature Sensing Yarns Positioned within the Temperature Sensing Fabric

**Figure 17:**
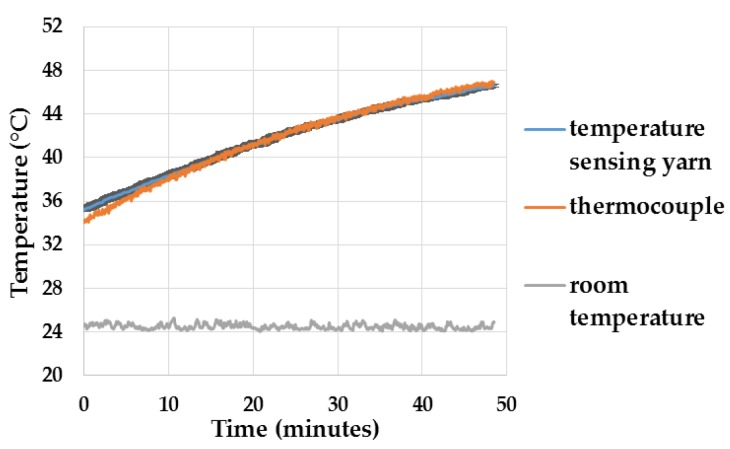
Temperature measurements when the temperature-sensing yarns were immersed in a beaker of water. The temperature recorded by the k type thermocouple is plotted in orange, the average temperature plotted by the temperature-sensing yarns are plotted in blue and the confidence intervals (95% confidence) for the temperature sensing yarns are plotted in black.

To understand how getting the system damp would affect the response of the temperature-sensing yarns, preliminary experiments were conducted where the temperature-sensing yarns were positioned within the temperature sensing fabric and immersed in beaker of water, as described in Section 2.9. The results as a function of time are presented below in Figure 17.

The results in Figure 17 show that the temperature recorded by the k type thermocouple lies exactly on top of the average temperature recorded by the three temperature-sensing yarns and its confidence intervals. The difference of 1.2 °C during the start of the experiment may have been caused due to the difference in response times of the sensors or as a result of the temperature of the water not being uniform. However, it can be concluded from the results that the moisture content would not impact the measurements of the temperature-sensing yarn. The Murata thermistor (NCP15XH103F03RC) when positioned in water stops functioning, hence it can be concluded that the polymer micro-pod protects the thermistor from moisture.

#### Identifying the Effects of Moisture Absorption and Evaporation

**Figure 18:**
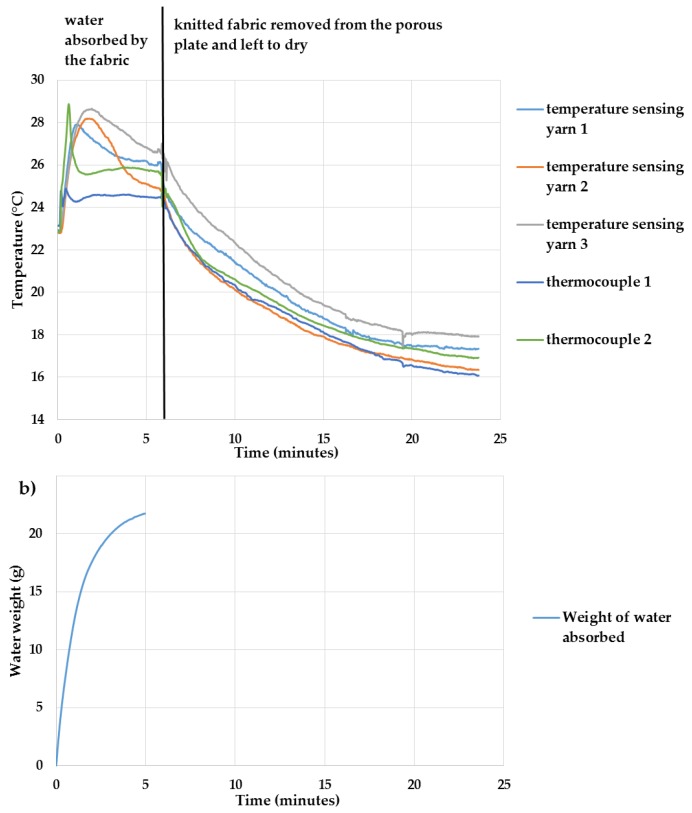
(**a**) Presents the temperature captured by the three temperature-sensing yarns and the thermocouples when the water was absorbed by the temperature-sensing fabric and when it is left to dry; (**b**) the rate at which the water absorbed by the temperature-sensing fabric.

The temperature measurements from the temperature-sensing yarns and the thermocouples when the temperature-sensing fabric absorbed water and when it was left to dry, is presented in Figure 18a. The rate of water absorbed by the temperature-sensing fabric was also recorded and it is given in Figure 18b.

As demonstrated in Figure 18a, the temperature rose when the warm water was absorbed by the knitted fabric. The temperature then decreased, which was likely due to the water evaporating and cooling the temperature sensing fabric. The difference between the measurements from different temperature-sensing yarns are likely due to different rates of absorption at different locations of the temperature sensing fabric. A detailed analysis was not conducted to identify the reason for these differences since the absorption and transportation of liquids within textiles are a highly complex processes which is dependent on many factors including temperature, humidity, airflow, fibre type and fibre density [48]; this did not fall within the scope of this work. The thermocouple measurements followed the same general trend as the measurements of the temperature-sensing yarn. The difference in-between the thermocouple measurements and the measurements from the temperature-sensing yarns might be due to there being no fibres in the channels containing the thermocouples. This would result in the absence of wicking in these channels and, therefore, it can be argued that the water did not properly wick through the channels containing the thermocouples.

Finally, the knitted fabric was taken away from the porous plate, and the temperature captured by both the temperature-sensing yarns and the thermocouples were seen to decrease as illustrated in Figure 18a. This was most likely caused due to evaporation.

As expected, the polymer micro-pod appears to have protected the thermistors in the temperature-sensing yarns from moisture. Therefore, it can be established that the temperature-sensing yarns could be used in wet or moist conditions.

### 3.8. Identifying the Effects of Compression, Bending and Stretching on the Temperature Sensing Fabric

The temperature measurements obtained from the temperature-sensing yarns when the temperature sensing fabric was left to rest and be compressed, bent and stretched are presented in Table 1.

The table above illustrates that compression, bending and stretching have no impact on the temperature measurements of temperature-sensing fabric. The average temperature measurements obtained during the four experiments fall within the accuracy range of the thermistor.

## 4. Conclusions

This work has successfully created and characterised a textile thermograph. Experiments have been conducted to fully understand the limitations of practically using such a device over a range of possible operational scenarios, and provides useful new knowledge regarding textile-based temperature sensing.

Initially, the accuracy of the absolute temperature measurements of a refined temperature sensing yarn design was explored. The paper demonstrated that when using a thermally conductive resin (9-20801) to encapsulate the thermistor, and when a 0.87 mm diameter micro-pod was used, no significant effect in the absolute temperature reading was observed over a physiologically relevant range of temperatures. When the diameter of the micro-pod was increased to 1.53 mm it introduced a measurement error of 0.05 ± 0.057; therefore, it was advisable to use a 0.87 mm diameter micro-pod. When this design was used to create a temperature-sensing yarn it was identified that the inclusion of packing fibres and the knit braided fibre sheath resulted in a measurement error of 0.12 ± 0.006.

The importance of proper contact between the temperature-sensing yarn and the surface being measured was characterised. In order to obtain highly accurate or relative measurements the temperature sensing-yarn has to be in contact with the surface being measured, even a 0.5 mm gap between the temperature-sensing yarn and the surface introduced a measurement error of 0.24 ± 0.03. Further, integrating the temperature-sensing yarn into a textile also introduces a measurement error of 0.24 ± 0.06, implying that this error was due to the distance between the yarns and the surface of the textile.

The impact of ambient temperature on the temperature captured by the temperature-sensing yarns was also demonstrated. A set of experiments were performed to identify the effects of covering and shielding the fabric containing the temperature-sensing yarns with different materials with varying thermal conductivities. The results have shown that using a thermally insulating material as the cover reduces the measurement error; however, using thermally conductive material as the cover increases measurement error, as might be expected. The next set of experiments illustrated that moisture content and relative humidity of the environment have no significant impact on the temperature measured by the temperature-sensing fabric. The final set of experiments have shown that bending, compression and stretching have no impact on the temperature measurements of the temperature-sensing fabric.

The work shows that obtaining highly accurate absolute temperature readings from a surface using a temperature-sensing yarn or a temperature-sensing fabric can be difficult if the surface temperature is lower or higher than the ambient temperature. The maximum difference in-between the absolute temperature and the temperature recorded by the temperature-sensing fabric was observed to be 2.54 ± 0.23 °C, for the physiologically relevant temperature range of 25–38 °C [42]. In order to obtain highly accurate measurements it is envisaged that the temperature-sensing yarn has to be calibrated according to the fabric it is integrated into, the ambient temperature and the insulation provided.

Lastly, the textile thermograph created functions as desired. Further work will include testing the thermograph in a pre-clinical setting.

## Figures and Tables

**Table 1:** Presents the average and standard deviation of the temperature measured by the temperature-sensing yarns in the fabric when the fabric was left to rest and be compressed, bent and stretched.

Rest	Compressed	Bent	Stretched
27.5 ± 0.4 °C	26.3 ± 0.4 °C	26.9 ± 0.3 °C	27.0 ± 0.3 °C
